# A Rare Association of Turner Syndrome, Celiac Disease, and Ulcerative Colitis in a Pediatric Patient: A Case Report

**DOI:** 10.3390/reports9040322

**Published:** 2026-09-23

**Authors:** Maria Rogalidou, Konstantina Dimakou, Kalliopi Stefanaki, Evanthia Botsa, Anna Messaritaki, Christina Kanaka-Gantenbein

**Affiliations:** 1 Division of Paediatric Gastroenterology, Hepatology & Nutrition, First Department of Pediatrics, “Agia Sofia” Children’s Hospital, Medical School, National & Kapodistrian University of Athens, Thivon & Papadiamantopoulou Street, Goudis, 11527 Athens, Greece; 2 Department of Gastroenterology, “Agia Sofia” Children’s Hospital, 11527 Athens, Greece; 3 Pathology Department, “Agia Sofia” Children’s Hospital, 11527 Athens, Greece; 4 First Department of Pediatrics, “Agia Sofia” Children’s Hospital, 11527 Athens, Greece; 5 Second Department of Pediatrics, “Agia Sofia” Children’s Hospital, 11527 Athens, Greece; 6 Division of Endocrinology, Diabetes and Metabolism, First Department of Pediatrics, “Agia Sofia” Children’s Hospital, Medical School, National & Kapodistrian University of Athens, 11527 Athens, Greece

**Keywords:** Turner syndrome, celiac disease, ulcerative colitis, inflammatory bowel disease, bone mineral density, autoimmunity, growth impairment

## Abstract

**Background and Clinical Significance**: Turner syndrome is associated with an increased prevalence of autoimmune disorders, particularly celiac disease, and is also associated with impaired bone health. The coexistence of Turner syndrome, celiac disease, and ulcerative colitis is rare and represents a challenging clinical scenario requiring multidisciplinary management; **Case Presentation:** We report the case of a 7-year-old girl referred for short stature who was diagnosed with celiac disease based on positive serology and duodenal histology (Marsh 3a/3c). Cytogenetic analysis revealed 46,X,i(X)(q10) Turner syndrome. Despite strict adherence to a gluten-free diet, persistent growth impairment led to initiation of recombinant human growth hormone therapy. At 14 years of age, she developed bloody diarrhea, weight loss, iron-deficiency anemia, and elevated inflammatory markers. Colonoscopy and histology confirmed extensive ulcerative colitis. During follow-up, dual-energy X-ray absorptiometry demonstrated reduced bone mineral density (lumbar spine Z-score −2.7; whole-body Z-score −2.5). At 16 years of age, treatment with azathioprine and 5-aminosalicylic acid was complicated by pancreatitis, with recurrence of pancreatic enzyme elevation following sequential drug reintroduction, leading to discontinuation of both agents. Infliximab therapy was subsequently initiated, resulting in sustained clinical, endoscopic, and near-complete histological remission. The patient was later transitioned to adult multidisciplinary care; **Conclusions:** The coexistence of Turner syndrome, celiac disease, ulcerative colitis, and low bone mineral density is an exceptionally rare combination of genetic susceptibility, autoimmunity, and chronic inflammation. This case highlights the importance of lifelong multidisciplinary surveillance in patients with Turner syndrome, including monitoring for additional autoimmune disorders, growth impairment, and skeletal complications.

## 1. Introduction and Clinical Significance

Turner syndrome (TS) is a chromosomal disorder caused by complete or partial absence of one X chromosome, affecting approximately 1 in 2500 live-born females. Beyond its classical features, including short stature and gonadal dysgenesis, TS is associated with an increased risk of autoimmune disorders and multisystem complications, including impaired bone health. Current clinical guidelines emphasize the importance of lifelong multidisciplinary surveillance and management of individuals with TS [1,2].

Celiac disease (CD) is among the most frequently reported autoimmune disorders in patients with TS. A recent systematic review and meta-analysis reported a pooled CD prevalence of approximately 4.5% in individuals with TS, supporting regular screening and early diagnosis to prevent complications such as growth impairment and reduced bone mineral density [3]. The mechanisms underlying the increased susceptibility to autoimmune disease in TS are not fully established, although abnormalities of X-chromosome dosage and immune regulation have been proposed as potential contributing factors [4]. Both TS and CD may independently contribute to impaired growth and compromised bone health, requiring careful monitoring throughout childhood and adolescence [1,3].

Inflammatory bowel disease (IBD), including ulcerative colitis (UC), has been reported less frequently in patients with TS. Although the coexistence of TS, CD, and UC is uncommon, it has been previously described in the literature [4,5]. The features of each medical condition, as well as the features common to all three conditions, are illustrated in Figure 1. The present case is notable for the combination of these conditions: genetic susceptibility, multiple immune-mediated diseases, chronic intestinal inflammation, and skeletal involvement represent a complex clinical scenario requiring coordinated multidisciplinary care.

We report the case of a girl with TS and CD who subsequently developed extensive UC, complicated by reduced bone mineral density and drug-induced pancreatitis, highlighting the importance of long-term surveillance for autoimmune manifestations, growth impairment, skeletal health, and treatment-related complications in patients with TS.

## 2. Case Presentation

We present the case of an asymptomatic girl who, at 7 years of age, was referred to the Pediatric Endocrinology Unit for evaluation of short stature. Her weight was approximately at the 50th percentile, whereas her height was below the 3rd percentile.

Initial investigations, including complete blood count, biochemical profile, hormonal evaluation, and bone age assessment, were unremarkable. Serological screening for CD was positive, with tissue transglutaminase IgA (tTG-IgA) levels of 56 U/mL (positive > 20 U/mL) and endomysial antibodies (EMA-IgA) at a titer of 1:160. Upper gastrointestinal endoscopy with duodenal biopsies confirmed CD (Marsh 3a and 3c). Concurrent cytogenetic analysis revealed a 46,X,i(X)(q10) karyotype, confirming the diagnosis of TS.

A strict gluten-free diet was initiated, with excellent adherence. However, despite normalization of celiac serology and continued dietary compliance, no significant catch-up growth was observed. Given the persistent growth impairment and the established indication for growth hormone therapy in TS, recombinant human growth hormone (rhGH) was initiated at age 8 years in accordance with Turner syndrome management guidelines, and continued until age 16 years, when epiphyseal closure was documented. The patient received rhGH at a dose of 12 mg/week, resulting in progressive improvement in linear growth with height improving from below the 3rd percentile at age 7 years to approximately to the 3rd percentile by age 16 years, as illustrated in Figure 2.

At 14 years of age, while maintaining a strict gluten-free diet and receiving recombinant human growth hormone (rhGH) therapy, she presented with bloody diarrhea, an approximately 3-kg weight loss, pallor, and laboratory findings consistent with hypochromic microcytic iron-deficiency anemia. Inflammatory markers and fecal calprotectin levels were markedly elevated.

At diagnosis, the patient had moderate-to-severe pancolitis, with a Mayo endoscopic score of II and a Pediatric Ulcerative Colitis Activity Index (PUCAI) score of 50. Laboratory evaluation demonstrated significant inflammatory activity, with a fecal calprotectin level of approximately 1000 μg/g, an erythrocyte sedimentation rate (ESR) of 57 mm/h, a C-reactive protein (CRP) level of 67 mg/L, and an albumin level of 3.0 g/dL. CMV PCR in intestinal biopsy was negative. Ileocolonoscopy demonstrated extensive pancolitis suggestive of inflammatory bowel disease, and histopathological examination confirmed the diagnosis of UC. Upper gastrointestinal endoscopy revealed mild gastritis. Magnetic resonance enterography (MRE) showed no evidence of small-bowel involvement but demonstrated diffuse colonic wall thickening, consistent with UC.

Intravenous corticosteroid therapy was initiated at 40 mg/day, resulting in clinical improvement, followed by transition to oral corticosteroids, which were gradually tapered and discontinued. Oral mesalamine was administered at 50 mg/kg/day. Following TPMT evaluation, azathioprine was initiated and gradually increased to 2 mg/kg/day. During subsequent treatment with azathioprine and 5-ASA, the patient developed pancreatitis. Appropriate medical therapy was initiated according to current guidelines [6].

One year after diagnosis, the patient developed a severe UC relapse with bloody diarrhea, weight loss, and anemia (hemoglobin, 9.5 g/dL). During hospitalization, she developed vomiting and abdominal pain with marked elevations in serum amylase (622 U/L), urinary amylase (6900 U/L), and lipase (1400 U/L), with ultrasonographic findings consistent with acute pancreatitis. Mesalamine and azathioprine were discontinued. Extensive investigations for autoimmune and viral causes were negative, and pancreatic enzymes normalized after drug withdrawal, supporting drug-induced pancreatitis.

Because of persistent UC activity (Mayo endoscopic score II; PUCAI, 70), corticosteroids were initiated. Rechallenge with mesalamine resulted within several days in recurrent epigastric pain, nausea, and elevated amylase (355 U/L) and lipase (763 U/L), which normalized after discontinuation. Mesalamine was therefore permanently discontinued. Infliximab was initiated, but because of an inadequate initial response, azathioprine was subsequently added. This resulted in asymptomatic pancreatic enzyme elevation, leading to permanent discontinuation of azathioprine. Thus, mesalamine-induced pancreatitis was confirmed by reproducible dechallenge–rechallenge, while azathioprine was associated with recurrent asymptomatic hyperenzymemia.

During follow-up, dual-energy X-ray absorptiometry (DEXA) demonstrated reduced bone mineral density, with a lumbar spine Z-score of −2.7 and a whole-body Z-score of −2.5, using age- and sex-specific reference values. Given the coexistence of Turner syndrome (TS), celiac disease (CD), chronic inflammatory disease, and corticosteroid exposure, all of which may adversely affect bone health, appropriate management of the low bone mineral density was initiated.

At 16 years of age, rhGH therapy was discontinued following epiphyseal closure. At follow-up, the patient achieved sustained clinical and endoscopic remission, with near-complete histological remission, while maintaining strict adherence to a gluten-free diet.

At the last follow-up visit, at 18 years of age, she was receiving infliximab monotherapy based on drug levels (10 mg/kg every 6 weeks). Her weight was 50.3 kg and height 155 cm (BMI, 20.9 kg/m^2^). Laboratory evaluation showed a hemoglobin level of 13.3 g/dL, ferritin 30 ng/mL, serum albumin 4.5 g/dL, and fecal calprotectin 75 µg/g. CRP was negative, while the ESR was 30 mm/h. Amylase 120 U/L, tTG and EMA results were negative.

Following transition from pediatric to adult services, she was transferred to adult multidisciplinary care and continues regular follow-up with gastroenterology and endocrinology specialists.

## 3. Discussion

TS is a chromosomal disorder caused by complete or partial absence of one X chromosome. Although the most common karyotype is 45,X, structural abnormalities of the X chromosome, including isochromosome Xq [46,X,i(X)(q10)], may occur. In our patient, TS was confirmed by cytogenetic analysis demonstrating a 46,X,i(X)(q10) karyotype. TS exclusively affects females and is characterized primarily by short stature and gonadal dysgenesis. Individuals with TS are also at increased risk of autoimmune disorders, cardiovascular abnormalities, metabolic complications, and reduced bone mineral density. The principal clinical manifestations of TS are summarized in Table 1 [2].

Autoimmune diseases are among the most important comorbidities in TS, with a lifetime prevalence approaching 60% [4]. CD is one of the most common autoimmune disorders in this population, with a prevalence substantially higher than that of the general population [3]. Consequently, current international guidelines recommend routine screening for CD in all girls with TS, even in the absence of gastrointestinal symptoms [1]. In our patient, CD was diagnosed during evaluation of short stature, highlighting the value of systematic screening. Although strict adherence to a gluten-free diet resulted in normalization of celiac serology, linear growth remained suboptimal, and rhGH therapy was initiated in accordance with current TS recommendations [1].

Seven years after the diagnosis of CD, our patient developed UC, illustrating the ongoing risk of additional immune-mediated disease in individuals with TS. Although the association between TS and CD is well established, the coexistence of TS, CD, and UC has only rarely been reported [5]. Immune dysregulation related to X-chromosome abnormalities and shared autoimmune pathways has been proposed as a potential mechanism linking these conditions [2,4]. Current ESPGHAN/ECCO guidelines emphasize early diagnosis, careful monitoring, and timely escalation of therapy in children with UC to achieve sustained remission and prevent long-term complications [6].

Reduced bone mineral density in our patient was likely multifactorial. TS itself predisposes to impaired bone accrual because of estrogen deficiency, altered bone geometry, and intrinsic skeletal abnormalities [1,7]. In addition, CD and chronic intestinal inflammation due to UC may further impair bone mineralization through malabsorption, nutritional deficiencies, and inflammatory cytokine-mediated bone loss [6,8]. Current TS guidelines recommend assessment of bone health during adolescence and continued surveillance in patients with additional risk factors. Similarly, ESPGHAN recommendations support assessment of bone health in children with CD who have persistent bone disease or additional risk factors for reduced bone mineral density [1,8]. In our patient, the coexistence of three conditions potentially affecting skeletal health supported the decision to perform DEXA assessment and initiate appropriate management.

Management was further complicated by suspected drug-induced pancreatitis during treatment with azathioprine and 5-ASA. Sequential rechallenge resulted in recurrent elevation of pancreatic enzyme levels, supporting intolerance to both medications. Escalation to infliximab subsequently resulted in sustained clinical and endoscopic remission with near-complete histological healing, consistent with current therapeutic approaches for pediatric UC when conventional therapies cannot be continued [6].

Although the coexistence of TS, CD, and UC has been previously reported, this combination remains exceptionally uncommon in clinical practice. The present case is therefore clinically noteworthy and contributes to the limited body of literature describing the coexistence of these three conditions. This case demonstrates the exceptionally rare coexistence of TS, CD, and UC, together with reduced bone mineral density and treatment-related pancreatitis. It underscores the complex interaction between genetic susceptibility, immune dysregulation, chronic intestinal inflammation, nutritional factors, and medication-related adverse events. Lifelong multidisciplinary surveillance is therefore essential in patients with TS, particularly those who develop multiple autoimmune and gastrointestinal conditions [1,2].

## 4. Conclusions

The coexistence of TS, CD, and UC is exceptionally rare and may be further complicated by reduced bone mineral density and treatment-related adverse events such as drug-induced pancreatitis. This case emphasizes the importance of routine screening for CD in girls with TS, continued vigilance for additional autoimmune and inflammatory disorders, and regular assessment of bone health in patients with multiple risk factors. Early recognition and coordinated management involving pediatric endocrinologists, gastroenterologists, dietitians, and transition services are essential to optimize growth, disease control, skeletal health, and long-term outcomes.

## Figures and Tables

**Figure 1:**
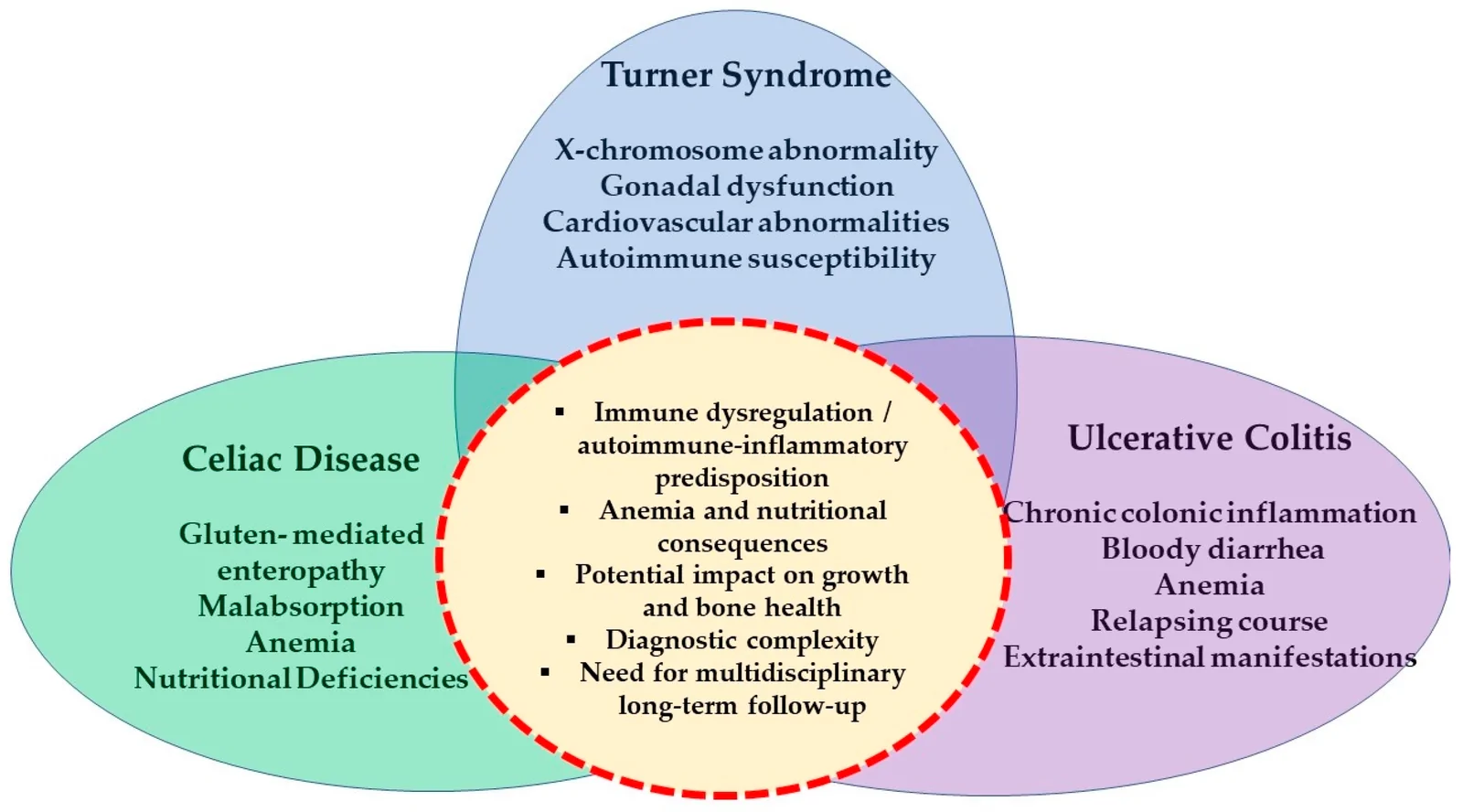
Venn diagram illustrating the clinical and pathophysiological overlap among Turner syndrome, celiac disease, and ulcerative colitis. Turner syndrome is associated with short stature and increased susceptibility to autoimmune disorders, including celiac disease. Celiac disease may contribute to malabsorption, nutritional deficiencies, and impaired bone health, whereas ulcerative colitis contributes to chronic intestinal inflammation, anemia, nutritional compromise, and systemic inflammatory burden. The overlapping areas highlight shared autoimmune and inflammatory susceptibility, potential gastrointestinal and nutritional consequences, and the cumulative impact of these conditions on bone health and long-term clinical management. The central intersection emphasizes the need for individualized, multidisciplinary follow-up. The diagram illustrates clinical coexistence and potential interactions and does not imply a direct causal relationship among the three conditions.

**Figure 2:**
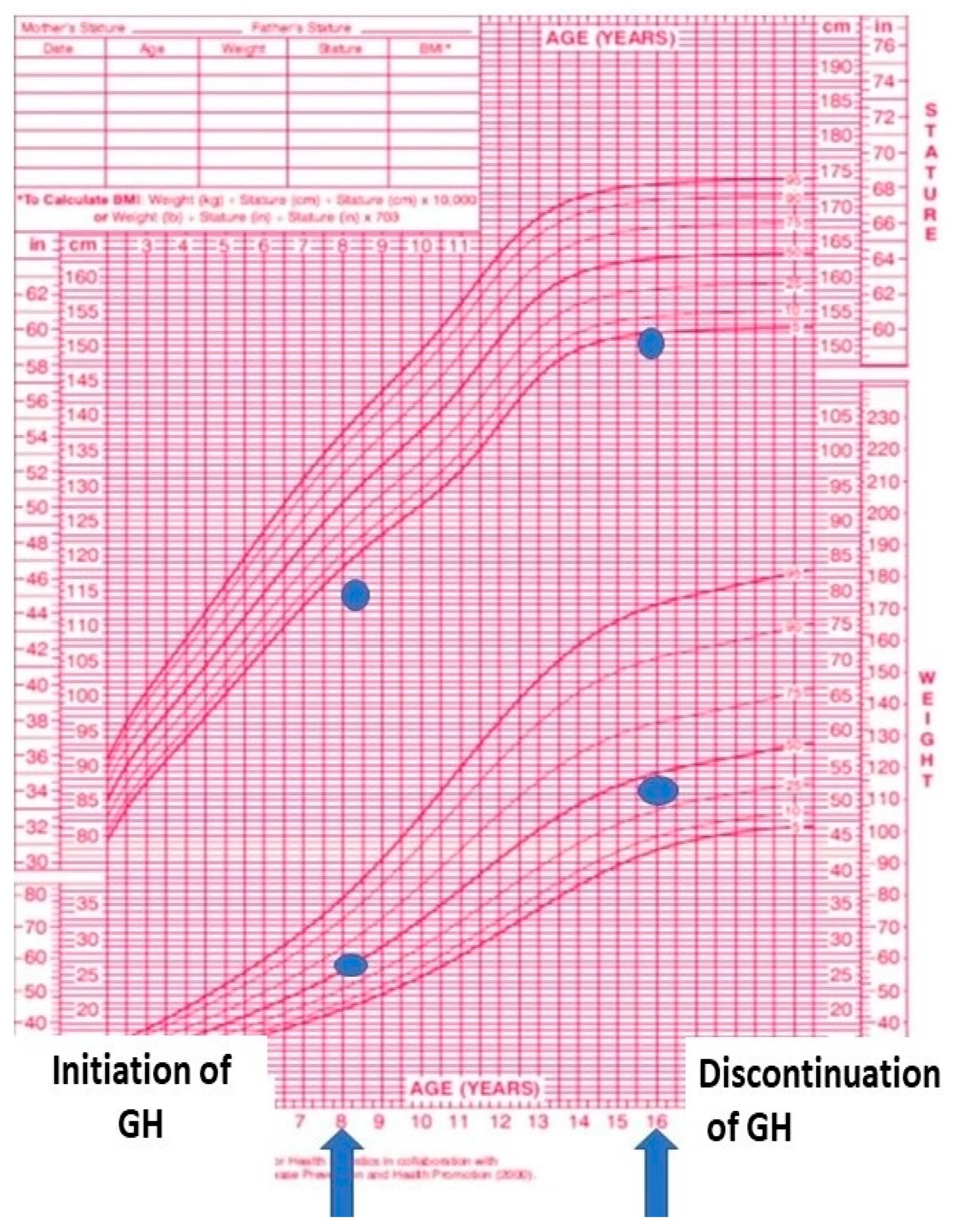
Growth chat of patient. Arrows show the age of initiation and discontinuation of GH, and bullets the weight and height of patient at the respective age.

**Table 1:** Turner Syndrome: Clinical manifestations.

Turner Syndrome	Clinical Manifestations	Findings in Our Patient
Physical Features	▪ Short stature (the most common clinical finding) ▪ Webbed neck (pterygium colli) ▪ Low posterior hairline ▪ Broad chest with widely spaced nipples ▪ Lymphedema of the hands and feet at birth ▪ Cubitus valgus (increased carrying angle of the elbows)	Yes Yes
Gastrointestinal Manifestations	▪ Increased risk of iron-deficiency anemia ▪ Increased risk of gastrointestinal bleeding ▪ Higher prevalence of celiac disease ▪ Increased frequency of inflammatory bowel disease	Yes Yes Yes (UC)
Hepatic Manifestations	▪ Frequent abnormalities in liver enzymes (elevated transaminases and/or cholestatic enzymes) ▪ Metabolic dysfunction-associated steatotic liver disease (MASLD) ▪ Autoimmune hepatitis ▪ Primary biliary cholangitis (PBC) ▪ Less commonly, vascular or structural abnormalities of the liver	
Renal Abnormalities	▪ Horseshoe kidney anomaly ▪ Other congenital anomalies of the urinary tract	
Autoimmune Disorders	▪ Lifetime prevalence of approximately 60% ▪ Celiac disease (prevalence approximately 4–7%) ▪ Autoimmune thyroid disease (most commonly Hashimoto thyroiditis) ▪ Vitamin B12 deficiency/pernicious anemia ▪ Vitiligo ▪ Addison’s disease (primary adrenal insufficiency) ▪ Psoriasis ▪ Lichen sclerosus	Yes
Endocrine and Metabolic Disorders	▪ Autoimmune thyroid disease ▪ Increased risk of type 1 diabetes mellitus ▪ Obesity and insulin resistance	
Cardiovascular Abnormalities	▪ Coarctation of the aorta ▪ Bicuspid aortic valve ▪ Dilatation of the ascending aorta ▪ Risk of aortic aneurysm/dissection	
Gonadal Function and Reproductive Manifestations	▪ Gonadal dysgenesis with formation of streak ovaries ▪ Primary ovarian insufficiency ▪ Delayed or absent spontaneous puberty ▪ Primary amenorrhea ▪ Infertility	
Bone Health	▪ Low bone mineral density (osteopenia/osteoporosis) ▪ Increased fracture risk	Yes

## Data Availability

The original contributions presented in this study are included in the article. Further inquiries can be directed to the corresponding author.

## Abbreviations

The following abbreviations are used in this manuscript:

TS	Turner syndrome
CD	Celiac disease
IBD	Inflammatory bowel disease
UC	Ulcerative Colitis
tTG	Tissue Transglutaminase
EMA	Endomysial antibodies
IgA	Immunoglobulin A
rhGH	Recombinant human growth hormone
PUCAI	Pediatric Ulcerative Colitis Activity Index
ESR	ESR erythrocyte sedimentation rate
CRP	C-reactive protein
MRE	Magnetic resonance enterography
DEXA	Dual-energy X-ray absorptiometry
5-ASA	5-aminosalicylic acid
ESPGHAN	European Society for Paediatric Gastroenterology, Hepatology and Nutrition
ECCO	European Crohn’s and Colitis Organisation
